# Expert perspectives on the nociplastic pain descriptor: a systematic review of textual evidence protocol

**DOI:** 10.11124/JBIES-26-00010

**Published:** 2026-07-09

**Authors:** Jelle C. Schouten, Wichor M. Bramer, Gwendolyne G.M. Scholten-Peeters, Frank J.P.M. Huygen, Jitske Tiemensma

**Affiliations:** 1 Department of Pain Medicine, Erasmus Medical Center, Rotterdam, The Netherlands; 2 Medical Library, Erasmus Medical Center, Rotterdam, The Netherlands; 3 Faculty of Behavioural and Movement Sciences, Amsterdam Movement Sciences - Program Musculoskeletal Health, Vrije Universiteit Amsterdam, Amsterdam, The Netherlands

**Keywords:** criticism, endorsement, nociplastic pain descriptor, systematic review, textual evidence

## Abstract

**Objective:**

The objective of this review is to systematically assess and synthesize textual evidence regarding the concept, including the terminology and definition, and International Association for the Study of Pain (IASP) criteria of the nociplastic pain descriptor.

**Introduction:**

Since its formal recognition by the IASP in 2017, the nociplastic pain descriptor has elicited both endorsement and critique. Currently, no systematic overview critically synthesizes the diverse expert perspectives on the nociplastic pain descriptor, contributing to misinterpretation, conceptual confusion, clinical challenges, and ongoing debate.

**Eligibility criteria:**

This review will include published and unpublished textual evidence reflecting expert perspectives on the nociplastic pain descriptor, including clinician narratives, expert opinion, and policy papers. Textual evidence on all body systems and any pain duration will be eligible, without restrictions based on contextual factors. Social media and video content will be excluded.

**Methods:**

This review will follow the JBI methodology for systematic reviews of textual evidence. Published textual evidence in any language from 2016 onward will be searched in Embase, MEDLINE, CINAHL, Web of Science, Scopus, and PsycINFO. Unpublished textual evidence will be searched for in specialized gray literature databases, Google, and international pain organization websites. Methodological quality will be assessed using the JBI critical appraisal checklists for textual evidence. Study selection, appraisal, data extraction, and synthesis will be conducted independently by 2 reviewers. Data synthesis will follow a meta-aggregative approach. Confidence in the findings will be graded using a modified ConQual approach. Good practice statements will be issued, where appropriate.

**Review registration:**

PROSPERO CRD420251270659

## Introduction

Chronic pain is a worldwide major health and socioeconomic problem, impacting quality of life, work, and everyday functioning.^1^ Current treatments show limited overall effectiveness, often benefit unidentified patient subgroups, and contribute to prolonged suffering and higher costs.^2^ Patient heterogeneity may obscure positive outcomes in specific subgroups. Precision medicine, which has already been implemented in disciplines such as cardiology and neurology, presents a promising approach by tailoring treatment based on patient characteristics such as risk factors or underlying biological mechanisms, thereby facilitating personalized treatment.^3^,^4^ This approach aims to improve outcomes and reduce costs, with mechanistic pain descriptors playing a key role in tailoring treatment.^2^

**Table:** Paper characteristics

Paper ID	Author(s)	Year	Publication status^a^	Types of papers^b^	Profession^c^	Country^d^	Reviewer summary	Notes
								
								

^a^ published or unpublished papers.

^b^ clinician narratives, expert opinion, or policy papers.

^c^ lead author profession.

^d^ lead author affiliation country.

**Table:** Extracted conclusions

Paper ID	Finding ID	Verbatim conclusion	Illustration	Page no.^a^	Credibility^b^	Domain^c^	Stance^d^	Notes
								
								

^a^ page number of the illustration.

^b^ unequivocal, credible, or not supported.

^c^ concept, terminology, definition, or IASP criteria.

^d^ criticism, endorsement, neutral.

The International Association for the Study of Pain (IASP) initially classified pain into 2 mechanistic descriptors: nociceptive and neuropathic pain.^5^ Nociceptive pain is defined as “pain that arises from actual or threatened damage to non-neural tissue and is due to the activation of nociceptors.”^6^^(para.24)^ In 2011, the IASP accepted a revised definition of neuropathic pain: “pain caused by a lesion or disease of the somatosensory nervous system,”^6^^(para.18)^ replacing the previously used word “dysfunction” with “disease.”^5^ This redefinition required evidence of a somatosensory lesion or disease, excluding many patients whose pain was neither neuropathic nor nociceptive. Moreover, the previous binary pain taxonomy failed to reflect the current understanding that chronic pain can be considered a disease in its own right, rather than merely a symptom.^7^ Additionally, the lack of a third descriptor led to stigmatizing labels such as idiopathic or dysfunctional pain.^5^ To address these clinical and conceptual limitations, and to improve the validation and communication of patients’ pain among patients, clinicians, and researchers, the IASP formally adopted nociplastic pain as a third mechanistic descriptor in 2017.^5^,^6^

Nociplastic pain is defined as “pain that arises from altered nociception despite no clear evidence of actual or threatened tissue damage causing the activation of peripheral nociceptors or evidence for disease or lesion of the somatosensory system causing the pain.”^6^^(para.27)^ Its underlying mechanisms remain poorly understood, but prolonged sensitization, both peripheral and central, is assumed to contribute to the altered nociception.^8^ Specifically, changes in cerebral activation, connectivity, and morphology associated with nociceptive function are observed in patients with chronic primary pain, alongside glial cell activation of the immune system.^5^,^8^,^9^

Clinically, nociplastic pain is characterized by regional, multifocal, or widespread pain with allodynia in the affected area, which lasts over 3 months and is not fully explained by pain mechanisms underlying nociceptive and/or neuropathic descriptors alone.^9^,^10^ It is often accompanied by a history of multimodal sensory hypersensitivity and comorbidities, such as sleep disturbances, fatigue, and cognitive problems. From a taxonomic perspective, nociplastic pain is associated with chronic primary pain diagnoses in the International Classification of Diseases 11th Revision (ICD-11), such as fibromyalgia.^7^,^9^ This descriptor, along with nociceptive and neuropathic pain, should be viewed as part of a chronic pain continuum in which mechanisms typically associated with different descriptors may overlap, often referred to as mixed pain.^2^,^5^,^9^

Since its formal adoption in 2017, research on the nociplastic pain descriptor has been expanding,^8^,^11^ focusing on discriminating it from other pain descriptors,^12^ developing the IASP clinical criteria and grading system,^10^ and field testing.^12^ The descriptor has gained acceptance in specialties such as rheumatology, gynecology, and oncology.^11^,^13^ However, the introduction of this descriptor has also elicited criticism from experts regarding its concept,^14^ terminology,^15^ definition,^16^ and IASP clinical criteria and grading system.^17^ This ongoing scientific debate, potentially fueled by evidence gaps in the foundational elements of the nociplastic pain descriptor, contributes to misinterpretation, conceptual confusion, and clinical challenges.

Given this ongoing debate, a systematic review would provide a structured method to synthesize textual evidence and identify gaps that can guide research, practice, and policy.^18^,^19^ Textual evidence includes documented communication sources beyond empirical studies, such as clinicians’ tacit knowledge and the prevailing health care discourse, informing decision-making. Research questions regarding concepts, including terminology and definitions, are best addressed with textual evidence, as they involve language, meaning, or theoretical framing not captured by quantitative studies. Although evidence gaps regarding the IASP criteria could be studied quantitatively (eg, reliability and diagnostic accuracy),^12^,^20^ a textual evidence review complements such evidence by focusing on interpretation and application rather than empirical properties. Accordingly, such a review can reveal inconsistencies, recurring concerns, and assumptions that lack empirical support, thereby identifying evidence gaps and highlighting priorities for future research.

A preliminary search on March 31, 2026, across PROSPERO, PubMed, *JBI Evidence Synthesis*, and the Cochrane Database of Systematic Reviews identified no published systematic reviews synthesizing textual evidence related to the nociplastic pain descriptor. However, 2 in-progress systematic reviews were identified.^21^,^22^ The first review will examine the pathophysiology and treatment effectiveness of centralized pain syndromes, using primarily empirical evidence and clinical outcomes,^21^ whereas our review will focus on nociplastic pain descriptor perspectives, using solely textual evidence to explore conceptual outcomes. The second review will synthesize textual evidence from reviews on the terminology and rationale for medically unexplained symptoms,^22^ whereas our review will synthesize textual evidence on the nociplastic pain descriptor, covering concepts, including terminology and definitions, and criteria, from both primary and secondary sources.

Given these limitations in the current literature, our systematic review will aim to inform practice and policy by providing a systematic overview of supportive and critical perspectives on the nociplastic pain descriptor and appraising their credibility, thereby supporting clinicians in the identification of nociplastic pain and more personalized care. It also will aim to inform future research by identifying evidence gaps.

## Review questions

What is currently known, contested, and uncertain regarding the nociplastic pain descriptor, based on expert perspectives?

Secondary research questions:

- What has been reported concerning the concept of the nociplastic pain descriptor, including its terminology and definition?
- What has been reported concerning the IASP clinical criteria and grading system of the nociplastic pain descriptor?

## Eligibility criteria

### Participants

This systematic review will consider expert perspectives on the nociplastic pain descriptor provided by clinicians and researchers as presented in clinician narratives, expert opinions, and policy papers. Eligible clinicians are medical or allied health care professionals with at least 5 years of experience in chronic pain management (determined from publicly available profiles). Eligible researchers are those with at least 1 peer-reviewed publication in this field, with eligibility in multi-author papers limited to first or last authors. These eligibility thresholds were determined by the research team to balance comprehensiveness with the experience needed to provide a meaningful perspective on the review questions.

### Phenomenon of interest

Perspectives on the nociplastic pain descriptor, as defined by the IASP, addressing its concept (including terminology and definition) and the associated IASP clinical criteria and grading system will be included. Perspectives encompass both criticism and endorsement. *Concept* refers to the theoretical framework, including proposed underlying mechanisms and its nature and meaning. *Terminology* concerns the appropriateness of the label “nociplastic,” while *definition* addresses the appropriateness of the definition.^6^ The *IASP criteria* include perspectives on their interpretation and application.^10^

Although the IASP criteria were developed for musculoskeletal conditions,^10^ papers on non-musculoskeletal chronic primary pain (eg, irritable bowel syndrome, endometriosis) will also be included, as the underlying mechanisms of nociplastic pain remain uncertain and may be relevant in these conditions.^11^ The criteria classify pain as nociplastic if it persists for over 3 months.^10^ Given the mechanistic uncertainties and to ensure a comprehensive overview, papers discussing its application to (sub)acute pain will also be included.

This review will focus on the nociplastic pain descriptor; papers using former labels will be included only if they discuss them in the context of nociplastic pain.

### Context

No restrictions will be applied to the inclusion of papers based on geographical, cultural, social, racial, temporal, or clinical setting factors. Expert perspectives on the nociplastic pain descriptor will be considered irrespective of the patient age group to which they refer.

### Types of evidence sources

In accordance with JBI methodology and our research question, we will include clinician narratives, expert opinions, and policy papers.^23^ Clinician narratives are accounts of clinical experiences with the nociplastic pain descriptor by clinicians. Expert opinions are views or statements from researchers or clinicians concerning the descriptor. Policy papers include documents guiding the descriptor from experts or professional associations.

To ensure comprehensive coverage and to reduce publication bias, this review will include both published and unpublished textual evidence. Published evidence encompasses letters to the editor, editorials, perspectives, viewpoints, commentaries, opinion pieces, position papers (including those from associations such as the IASP), debates, and narrative reviews. Unpublished evidence includes conference proceedings and abstracts, theses and dissertations, media reports, preprints, and web pages. Consistent with current recommendations, social media and video content are excluded due to challenges in identification and critical appraisal.^24^

## Methods

The proposed systematic review will be conducted following the JBI methodology for systematic reviews of textual evidence.^23^ The review will be reported in line with the Preferred Reporting Items for Systematic Reviews and Meta-Analyses (PRISMA) guidelines.^25^ This review has been registered in PROSPERO (CRD420251270659).

### Search strategy

Following JBI recommendations, a 3-step search strategy for published textual evidence was developed in collaboration with a medical librarian, who assisted in designing the sequential search steps and developing the database-specific search strings.^23^,^24^ We slightly modified the strategy by searching for the nociplastic pain descriptor in titles, abstracts, or author keywords in Embase, MEDLINE, CINAHL, Web of Science, Scopus, and PsycINFO, rather than broader keywords and indexing terms. We believe this modification was justified because nociplastic pain is a specific and emerging concept, and alternative terminology is unlikely to yield relevant literature for our review. We then expanded the search to papers on the classification of chronic pain, followed by letters and editorials on chronic pain classification and differences between nociceptive and neuropathic pain. To ensure comprehensive coverage, backward and forward citation tracking will be conducted using Web of Science for all included papers following full-text screening, where feasible. As discussions on the nociplastic pain descriptor began in 2016, papers published before 2016 will be excluded.^5^ No language restrictions will be applied. The full search strategy for Embase is provided in Appendix I.

Unpublished textual evidence will be searched through specialized databases, Google, and the websites of international pain organizations. Two reviewers (JS and JR) will independently search specialized gray literature databases, perform Google searches (in incognito mode), and manually check international pain organization websites for relevant sources.^23^ The full unpublished textual evidence search strategy is provided in Appendix II.

### Source of evidence selection

Following the search, all papers will be uploaded to EndNote v.21.2 (Clarivate Analytics, PA, USA), and duplicates removed using the method by Bramer *et al*.^26^ Source selection will occur in Covidence (Veritas Health Innovation, Melbourne, Australia). Papers not published in Dutch or English will be translated by native speakers within the research team’s network or, if needed, by professional translation services. Before screening, 2 reviewers (JS and JR) will independently pilot 25 randomly selected studies. If agreement is at least 75%, the screening will proceed; otherwise, discrepancies will be discussed to calibrate the application of the eligibility criteria, and this process will be repeated until the threshold is reached. Screening will begin with titles and, when available, abstracts, before moving on to full-text assessment. Two reviewers (JS and JR) will screen sources independently. Disagreements will be resolved by discussion or with a third reviewer (JT) when needed. Reasons for full-text exclusions will be recorded. The selection process will be reported using a PRISMA flow diagram and accompanying narrative.

### Quality assessment

Critical appraisal of textual evidence examines authenticity, credibility, logic of justification, and potential motives of the included papers.^23^ We will use the JBI critical appraisal checklists for narrative, expert opinion, and policy papers.^23^ These checklists assess multiple domains; credibility includes the author’s standing in the field, defined in this review as at least 5 years of clinical experience or 1 peer-reviewed publication in chronic pain management. For narrative reviews, we will use the Scale for the Assessment of Narrative Review Articles (SANRA), as JBI checklists are not suitable for assessing this type of evidence.^27^ Two reviewers (JS and JR) will assess the quality independently and resolve disagreements through discussion or by consulting a third reviewer (JT), if necessary. Before appraisal, reviewers will standardize their approach. A pilot test of 4 papers per text type will be conducted. If agreement is above 75%, full appraisal will proceed; otherwise, further discussion will ensure consistent application of scoring criteria.

Papers will not be excluded based on appraisal scores, ensuring a comprehensive overview of textual evidence. Results will be presented in tables and narrative summaries, detailing individual papers, quality domains, evidence types, and the overall dataset. As no accepted method exists for integrating appraisal scores into synthesis, these results will instead inform the narrative interpretation and discussion of the findings.

### Data extraction

Data will be extracted by 2 reviewers (JS and JR) independently using 2 modified JBI data extraction tools available in the JBI System for the Unified Management, Assessment and Review of Information (JBI SUMARI; JBI, Adelaide, Australia; Appendix III).^28^ Any disagreements will be resolved through discussion or with a third reviewer (JT), and a pilot extraction will be conducted on the first 3 papers. Authors will be contacted for additional information, when required. The data extraction will consist of 2 stages: i) recording paper characteristics, and ii) extracting authors’ verbatim conclusions and corresponding illustrations, and assessing credibility (unequivocal, credible, or not supported) based on reviewer judgment of the alignment between each conclusion and its supporting data.^23^ For narrative reviews, only the interpretative text of the review authors will be extracted. Text from primary studies will be considered separately.

### Data synthesis

Textual data will be synthesized using a meta-aggregation approach with JBI SUMARI.^23^,^28^ Findings will be reported narratively where pooling is not possible. Data for all types of papers will be synthesized as a single body of evidence and organized by domain (concept, terminology, definition, and IASP criteria), with only unequivocal or credible data included. Unsupported data will be reported in the extraction tables but excluded from the synthesis. The textual pooling process will involve 3 stages: i) extracting and appraising verbatim level 1 conclusions from full-text papers; ii) grouping these conclusions by meaning into categories to form level 2 conclusions; iii) synthesizing level 2 categories into level 3 conclusions, which represent the synthesized findings. Prior to synthesis, reviewers JS and JR will reach consensus on synthesis steps, with all decisions documented in the review manuscript.

### Assessing confidence in the findings

The final synthesized findings will be graded according to the ConQual approach for establishing confidence in the output of qualitative research synthesis and presented in a Summary of Findings.^29^ The dependability component of ConQual will not be applied, as it cannot be assessed in textual evidence papers that lack a research methodology. Although expert opinion typically starts at a low ConQual ranking in qualitative evidence synthesis, we will start at a high ranking because textual evidence is the most appropriate source for our review question. Separately, we will apply the Grading of Recommendations Assessment, Development and Evaluation (GRADE) criteria to determine whether issuing a good practice statement is appropriate.^30^ All 5 criteria will need to be met for a synthesized finding to be designated as a good practice statement, which is separate from standard GRADE recommendations.

## Acknowledgments

Bea Spek at the University of Amsterdam for her insightful lessons on qualitative research and her feedback on the protocol.

Paul Hodges at the University of Queensland for his contributions to the development of this protocol during the brainstorming session.

## Declarations

ChatGPT (OpenAI, San Francisco, USA; GPT-5-mini) was used for minor language refinement under the author’s supervision.

## Author contributions

JCS: conceptualization and writing of the manuscript; WMB: conceptualization and development of the search and source selection strategy; GGMS-P, FJPMH, JT: review and editing.

## Appendix I: Search strategy for published textual evidence

**Table:** Papers on nociplastic pain

Database	Query
Embase	(‘nociplastic pain’/de OR (nociplast* NEAR/3 pain*):ab,ti,kw)

**Table:** Papers on the classification of chronic pain

Database	Query
Embase	(‘nociceptive pain’/mj OR ‘neuropathic pain’/mj OR ‘chronic pain’/mj OR ((chronic* OR nocicept* OR neuropath*) NEAR/3 pain*):ti) AND (‘coding and classification’/mj OR ‘nomenclature’/mj OR classification/mj OR ‘clinical classification’/mj OR ‘disease classification’/mj OR ‘conceptual model’/mj OR conceptualization/de OR differentiation/mj OR definition/de OR ‘clinical criteria’/de OR ‘grading system’/de OR algorithm/mj OR (classif* OR nomenclat* OR categori* OR concept* OR framework* OR differentiat* OR terminolog* OR taxonom* OR definit* OR define* OR discriminat* OR (clinical* NEAR/3 criteria*) OR grading* OR algorithm* OR descriptor*):ti)

**Table:** Letters and editorials on the classification of chronic pain

Database	Query
Embase	(‘nociceptive pain’/de OR ‘neuropathic pain’/de OR ‘chronic pain’/de OR ((chronic* OR nocicept* OR neuropath*) NEAR/3 pain*):ab,ti,kw) AND (‘coding and classification’/de OR ‘nomenclature’/de OR classification/de OR ‘clinical classification’/de OR ‘disease classification’/de OR ‘conceptual model’/de OR conceptualization/de OR differentiation/de OR definition/de OR ‘clinical criteria’/de OR ‘grading system’/de OR algorithm/de OR (classif* OR nomenclat* OR categori* OR concept* OR framework* OR differentiat* OR terminolog* OR taxonom* OR definit* OR define* OR discriminat* OR (clinical* NEAR/3 criteria*) OR grading* OR algorithm* OR descriptor*):ab,ti,kw) AND ([letter]/lim OR [editorial]/lim OR (reply OR editorial* OR comment* OR letter* OR perspective* OR viewpoint* OR commentar* OR opinion-piece* OR position-paper* OR debate*):ti)

**Table:** Letters and editorials on the differences between nociceptive and neuropathic pain

Database	Query
Embase	(‘nociceptive pain’/de OR (nocicept* NEAR/3 pain*):ab,ti,kw) AND (‘neuropathic pain’/de OR (neuropath* NEAR/3 pain*):ab,ti,kw) AND ([letter]/lim OR [editorial]/lim OR (reply OR editorial* OR comment* OR letter* OR perspective* OR viewpoint* OR commentar* OR opinion-piece* OR position-paper* OR debate*):ti)

**Table:** Papers retrieved from the multi-step published textual evidence search strategy

Embase.com			
Search conducted: March 16, 2026			
Years of coverage	Records	2016 onward	Records after deduplication
1971–present	3244	2229	1081

No database limits were used other than those specified in the search strategies.

## Appendix II: Search strategy for unpublished textual evidence

**Table:**

Source or database	Website	Query or searching method
Google advanced search^a^	www.google.com/advanced_search	“nociplastic AND concept”
Google advanced search^a^	www.google.com/advanced_search	“nociplastic AND definition”
Google advanced search^a^	www.google.com/advanced_search	“nociplastic AND terminology”
Google advanced search^a^	www.google.com/advanced_search	“nociplastic AND criteria”
Google Scholar advanced search^a^_,_^b^	https://scholar.google.com/	“nociplastic AND concept”
Google Scholar advanced search^a^_,_^b^	https://scholar.google.com/	“nociplastic AND definition”
Google Scholar advanced search^a^_,_^b^	https://scholar.google.com/	“nociplastic AND terminology”
Google Scholar advanced search^a^_,_^b^	https://scholar.google.com/	“nociplastic AND criteria”
OpenGray Repository	https://lifesciences.datastations.nl/	Use search feature with query “nociplast*”
ProQuest Conference Papers Index	www.proquest.com/	Use search feature (Conference Papers & Proceedings) with query “nociplast*”
ProQuest Dissertations and Theses	www.proquest.com/	Use search feature (Dissertations & Theses) with query “nociplast*”
ProQuest Newspapers	www.proquest.com/	Use search feature (Newspapers) with query “nociplast*”
Turning Research Into Practice (TRIP)	www.tripdatabase.com/	Use search feature with query “nociplast*”
Website pain organization: International Association for the Study of Pain	www.iasp-pain.org/	Manual browsing of website and when search feature available use query “nociplastic”
Website pain organization: European Pain Federation	https://europeanpainfederation.eu/	Manual browsing of website and when search feature available use query “nociplastic”
WorldCat Dissertations and Theses	https://search.worldcat.org/nl	Use search feature with query “nociplast*”

^a^ first 10 pages of results.

^b^ search limited to papers from 2016 onward.
